# Comparing the odds of postpartum haemorrhage in planned home birth against planned hospital birth: results of an observational study of over 500,000 maternities in the UK

**DOI:** 10.1186/1471-2393-12-130

**Published:** 2012-11-19

**Authors:** Andrea Nove, Ann Berrington, Zoë Matthews

**Affiliations:** 1 Division of Social Statistics, University of Southampton, Southampton, England; 2 Southampton Statistical Sciences Research Institute and ESRC Centre for Population Change, University of Southampton, Southampton, England; 3 Centre for Global Health, Population and Poverty, University of Southampton, Southampton, England

**Keywords:** Home birth, Safety, Postpartum haemorrhage, Hospital birth

## Abstract

**Background:**

The aim of this study is to compare the odds of postpartum haemorrhage among women who opt for home birth against the odds of postpartum haemorrhage for those who plan a hospital birth. It is an observational study involving secondary analysis of maternity records, using binary logistic regression modelling. The data relate to pregnancies that received maternity care from one of fifteen hospitals in the former North West Thames Regional Health Authority Area in England, and which resulted in a live or stillbirth in the years 1988–2000 inclusive, excluding ‘high-risk’ pregnancies, unplanned home births, pre-term births, elective Caesareans and medical inductions.

**Results:**

Even after adjustment for known confounders such as parity, the odds of postpartum haemorrhage (≥1000ml of blood lost) are significantly higher if a hospital birth is intended than if a home birth is intended (odds ratio 2.5, 95% confidence interval 1.7 to 3.8). The ‘home birth’ group included women who were transferred to hospital during labour or shortly after birth.

**Conclusions:**

Women and their partners should be advised that the risk of PPH is higher among births planned to take place in hospital compared to births planned to take place at home, but that further research is needed to understand (a) whether the same pattern applies to the more life-threatening categories of PPH, and (b) why hospital birth is associated with increased odds of PPH. If it is due to the way in which labour is managed in hospital, changes should be made to practices which compromise the safety of labouring women.

## Background

### Introduction

Studies of the comparative safety of home and hospital birth have tended to focus on perinatal death as the main outcome measure, rather than the question of whether planned home birth is safe from the perspective of the mother’s wellbeing. This is understandable; if planned home birth is associated with a greatly elevated risk of serious negative infant outcomes, then most women and clinicians would be reluctant to attach as much importance to other benefits it might offer. A few recent studies have concluded that under some circumstances there is a small increased risk to the baby if the mother plans a home birth [1,2]. However, most recent research indicates that, from the point of view of the baby’s health and survival, planned home birth in developed countries can be as safe as planned hospital birth in low-risk pregnancies to parous women [3-8]. Perhaps, therefore, it is time for the safety of the mother to play a more central role in the debate. Indeed, it has been argued that, even if there was a small additional risk for the baby, the right of the mother to choose home birth on the grounds of her own safety could outweigh other considerations [9].

The ultimate measure of the safety of birth from the mother’s point of view is maternal mortality. However, in developed countries maternal death is now so rare that it would be very difficult to construct a dataset that would allow a valid comparison of the relative risk of maternal death in different birth settings. Instead, we must consider other maternal outcomes that have the potential to lead to maternal death or to serious maternal morbidity. Postpartum haemorrhage (PPH) has been identified by the UK Care Quality Commission as one of three “potential markers relating to the risk of maternal mortality” [10].

Previous research from the UK and Canada has identified a lower risk of PPH among planned home births than among planned hospital births [4,11], but the UK study did not attempt to control for confounding variables. Research from Australia has found no significant difference between planned home birth and hospital birth in terms of the risk of PPH [1].

Using a unique UK dataset, this paper addresses the question: ‘is the incidence of PPH different if a home birth was intended than if a hospital birth was intended?’ This is the first time that a UK-based study has attempted to answer this question using multivariable analysis techniques to control for known confounders such as: parity, anaemia, maternal age and maternal BMI [12,13]. The results will provide further evidence to help pregnant women, their partners and maternity care providers to make a more informed choice about place of birth than has been possible with previously available evidence.

## Methods

This is an observational study involving secondary analysis of maternity records, in which information was recorded contemporaneously by health professionals as pregnancies progressed. In the UK, even if a home birth is planned, a pregnant woman receives maternity care from health care professionals who are based at an individual hospital, so the hospital records included planned home births as well as planned hospital births. The study data were taken from the St Mary’s Maternity Information System (SMMIS), a computerised records system which was used by most of the hospitals within the former North West Thames Regional Health Authority (RHA) area during the study period. Between 1988 and 2000 (inclusive), 15 National Health Service (NHS) hospitals contributed data relating to all the pregnancies for which they provided any maternity care. The participating hospitals came from a wide range of types and locations, so there is no reason to suppose that the results are unrepresentative of the region as a whole.

A total of 585,291 pregnancies from the 15 hospitals were included in the SMMIS database. Studies have concluded that the completeness and quality of the information recorded within SMMIS is good. For example, studies comparing the information recorded on the database against case notes found a very high degree of corroboration (at least 95% agreement for most variables, but with somewhat lower levels of corroboration for maternal blood pressure and haemoglobin levels), and a high level of consistency across different hospitals [14,15].

**Figure 1:**
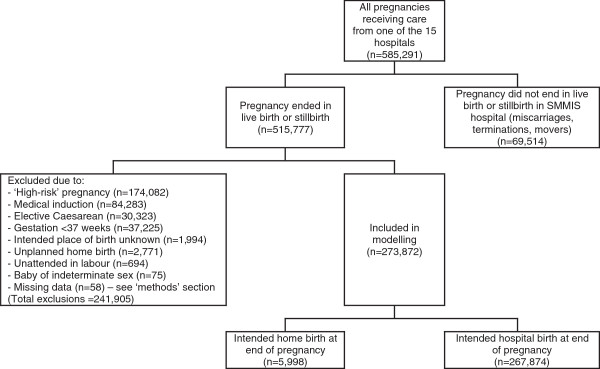
Numbers and types of pregnancies included in this analysis.

Figure 1 illustrates the groups excluded from this analysis. All pregnancies which did not end in either a live birth or stillbirth were excluded because they were not relevant to the research question, ie miscarriages and terminations were not part of the analysis. Pregnancies for which the intended place of birth was not known were also deleted (0.4% of the study population), because it was not possible to determine whether the place of birth was planned or unplanned. Because unplanned home births are known to have worse outcomes than planned home births and planned hospital births [16,17], this is a crucial distinction.

Unplanned home births were excluded from the analysis because they would have all been classed as having intended a hospital birth and their inclusion would have artificially increased the risks associated with planning a hospital birth. It could be argued that unplanned home births are similar to planned home births which were transferred to hospital during labour (because birth did not take place in the intended location), and that not getting to hospital in time is a risk of planning a hospital birth, and for this reason we have run the analysis both with and without unplanned home births (see ‘results’ section). However, there is a fundamental difference between the two situations: transferring from home to hospital generally involves a considered decision made by the labouring woman and her partner in consultation with the attending midwife. Unplanned home births do not involve a considered decision – they are an unavoidable response to circumstances such as very quick labour. Planning a home birth would not have avoided this risk completely, because there is still a chance that that the midwife would not have arrived in time for the delivery. By contrast, planning a hospital birth would generally avoid the risk of having to travel to hospital during established labour.

Research on the comparative safety of different birth settings tends to exclude ‘high-risk’ pregnancy; conventional wisdom states that women with ‘high-risk’ pregnancies should plan a hospital birth because they are at higher risk of negative pregnancy outcomes. In fact, there is little hard evidence to suggest that, if the pregnancy is ‘high-risk’, a negative outcome is more likely if a home birth is attempted, so there is an argument for including ‘high-risk’ pregnancies in this type of analysis, and this was attempted as part of this research project. The inclusion of ‘high-risk’ pregnancies in the analysis necessitated a significantly more complex model to control for the fact that the ‘intended hospital birth’ group contained a higher proportion of ‘high-risk’ pregnancies. Numerous conditions render a pregnancy ‘high-risk’, and the attempt to control for them all led to a model containing 27 covariates, which introduced the possibility of statistical problems such as collinearity. For this reason, the main results shown exclude ‘high-risk’ pregnancies. However, a model which included them was also fitted, with results given alongside the main findings for comparison.

Women who were classed as not having had a birth attendant were also excluded. These were mostly women who had intended a home birth and had had very rapid labours (the mean labour length for all those who intended a home birth was 6.0 hours, but among those who were unattended by an appropriate health professional it was 2.1 hours). Presumably in these cases the baby was delivered before the midwife arrived at the woman’s home. It was therefore possible to categorise them with unplanned home births in terms of the circumstances surrounding their labours and births. As with unplanned home births, an argument could be made for including unattended births in the analysis, as giving birth unattended is a risk of planning a home birth, so, as a further sensitivity analysis, the model was re-run with these observations included (see ‘results’ section).

Elective Caesarean sections were also excluded from the analysis. PPH was more common if the baby was delivered by Caesarean section than if born vaginally (in SMMIS, PPH occurred in 6.7% of emergency Caesareans and 4.3% of elective Caesareans, compared with just 1.1% of vaginal births). Because elective Caesareans occurred only in the ‘intended a hospital birth’ group, their inclusion in this analysis would have artificially inflated the risk of PPH for hospital births, because elective Caesareans tend to be performed in response to fears about the safety of vaginal delivery, eg if the foetus is malpresented. For similar reasons, medical inductions (ie those using oxytocin and/or prostaglandins) were excluded. These too only occurred in the ‘intended a hospital birth’ group, so that comparisons would have become irrelevant if the cases had been included.

### Definitions

There are a number of definitions of PPH. According to the Royal College of Obstetricians and Gynaecologists (RCOG), although an estimated blood loss of at least 500ml counts as a PPH, in the UK a case should be considered an “emergency” only when the blood loss exceeds 1000ml [18]. For this reason, the definition of PPH adopted for this analysis was the loss of at least 1000ml of blood. In both the ‘home’ and ‘hospital’ groups, the amount of blood lost was recorded by clinicians and later inputted into a field in the SMMIS database.

Women were classed as having intended a home birth if: (a) a home birth was intended at booking and the baby was delivered at home, (b) a hospital birth was intended at booking but the baby was delivered at home, and SMMIS recorded the change in intention as having taken place before labour commenced, or (c) a home birth was intended at booking but the baby was delivered in hospital, and SMMIS recorded the change in intention as having taken place during labour. Thus, intrapartum transfers from home to hospital were included in the ‘intended a home birth’ group. Women were classed as having intended a hospital birth if: (a) a hospital birth was intended at booking and the baby was delivered in hospital, or (b) a home birth was intended at booking but the baby was delivered in hospital, and SMMIS recorded the change in intention as having taken place before labour commenced. Maternities were classed as unplanned home births if a hospital birth was intended at booking but the baby was delivered at home, and SMMIS recorded the change in intention as having taken place during labour.

The risk status of a pregnancy was defined using a mixture of maternal International Classification of Disease (ICD) codes [19] and individual fields in the SMMIS database, and was based on a 2007 clinical guideline from the National Institute for Health and Clinical Excellence (NICE) which contained lists of medical and obstetric conditions which indicate increased risk of negative pregnancy outcomes [20]. Some were listed as “suggesting planned birth at an obstetric unit” and some as “indicating individual assessment when planning place of birth”. Pregnancies with conditions in the former list were classed as ‘high-risk’, and those with conditions in the latter list as ‘medium-risk’. All other pregnancies were classed as ‘low-risk’.

### Statistical analyses

The analysis was carried out using a logistic binary regression model, with PPH as the outcome variable and built using manual forward selection (with p < 0.05 as the cut-off). Because SMMIS contained over 200 items of information for each pregnancy, the list of potential covariates was a long one. Covariates were selected after a literature review of characteristics associated with intended place of birth [10,11,21-23] and/or PPH [12,13,18,24].

Some potential covariates were excluded from the modelling despite being associated with PPH: mode of delivery, type of health professional attending delivery, type of pain relief used in labour and augmentation of labour. This is because these factors may act as mediators and may explain the difference between home and hospital birth, and therefore holding them constant would have led to controlling for the effect of planned place of birth on PPH. Had the aim of this analysis been to identify characteristics associated with PPH, clearly these covariates would have been included (as would many of the maternities excluded from the analysis as described earlier), so it would not be appropriate to use these results to draw conclusions about the association between PPH and covariates other than intended place of birth.

A number of covariates were not included in the final model because, after adjustment for the other model covariates, there was no significant association between them and PPH. These covariates are listed in Table 1.

**Table 1:** Covariates excluded from model due to having no significant association with postpartum haemorrhage

	
Month of delivery	Chorionic villus biopsy in pregnancy
Congenital abnormality suspected in pregnancy	Amniocentesis in pregnancy
Actual congenital abnormality	Interpreter required
Booking appointment after 20 weeks gestation	Previous terminations
Mother’s Carstairs quintile [25] (a measure of deprivation)	Previous miscarriages
Maternal skeletal condition (eg previous fractured pelvis, spinal abnormality)	Fibroids
Borderline maternal hypertension (BP 140 systolic or 90 diastolic)	Maternal inflammatory bowel disorder
Mother’s smoking status	

Once the final additive model was built, interaction terms were tested, involving intended place of birth and: pregnancy risk factors, year, parity, maternal age and time of birth. None made a statistically significant improvement to the model fit.

There were no missing data for the outcome variable. The approach for handling missing data for the explanatory variables depended on the extent of the problem. If fewer than 0.1% of records had data missing on a variable, these records were deleted. If between 0.1% and 12% of records had data missing on a variable, a ‘missing’ category was created and included as a separate measure within the model. If more than 12% of records had missing data on a variable, that variable was not included as a covariate.

### Ethical approval

The Riverside Research Ethics Committee (REC) approved the project (REC reference number 08/H0706/42) on 17 April 2008.

## Results

Among the 273,872 pregnancies which were used for the analysis described in this paper, there were 2,808 cases of PPH (1.02% of the eligible records). In the unadjusted data, the incidence of PPH was significantly higher in the ‘intended a hospital birth’ group than in the ‘intended a home birth’ group (1.04% and 0.38% respectively – see Table 2). A chi-squared test showed that this difference was highly statistically significant (p = 0.000).

**Table 2:** Unadjusted incidence of postpartum haemorrhage (PPH), by characteristics of mother and pregnancy

	**No. of women in this group**	**No of PPHs in this group**	**% of women suffering PPH**
**Intended place of birth**			
Hospital	267,874	2,785	1.04
Home	5,998	23	0.38
Pregnancy risk status*			
Medium	73,862	968	1.31
Low	200,010	1,840	0.92
**Parity**			
Primipara	125,963	1,653	1.31
Multipara	147,909	1,155	0.78
**Mother’s age at delivery**			
<20	13,881	111	0.80
20-24	51,640	436	0.84
25-29	93,757	915	0.98
30-34	81,332	903	1.11
35-39	29,031	367	1.26
40+	4,231	76	1.80
**Mother’s ethnic group**			
Black African	7,516	130	1.73
Black Caribbean	6,587	79	1.20
Mediterranean	6,808	62	0.91
Oriental	4,350	72	1.66
South Asian	34,674	320	0.92
White European	195,498	1,940	0.99
Other	11,064	137	1.24
Missing	7,375	68	0.92
**Current baby’s birthweight**			
Low (<2500g)	5,122	31	0.61
2500g-3999g	241,301	2,195	0.91
4000g+	27,449	582	2.12
**Sex of baby**			
Boy	140,548	1,306	0.93
Girl	133,324	1,502	1.13
**Number of ultrasound scans in pregnancy**			
0	4,610	51	1.11
1	114,588	1,005	0.88
2	99,368	1,091	1.10
3	35,376	384	1.09
4	10,951	166	1.52
>4	5,748	84	1.46
Missing	3,231	27	0.84
**Year of delivery**			
1988	20,901	159	0.76
1989	21,939	187	0.85
1990	22,311	234	1.05
1991	22,108	189	0.85
1992	22,040	208	0.94
1993	21,077	186	0.88
1994	21,014	189	0.90
1995	20,066	228	1.14
1996	20,950	222	1.06
1997	20,246	239	1.18
1998	20,087	240	1.19
1999	20,267	263	1.30
2000	20,866	264	1.27
**Hospital providing care (anonymised)**			
A	8,620	73	0.85
B	16,969	117	0.69
C	7,958	66	0.83
D	21,167	321	1.52
E	8,177	88	1.08
F	20,041	192	0.96
G	20,784	150	0.72
H	25,066	237	0.95
I	29,819	389	1.30
J	22,954	298	1.30
K	19,940	228	1.14
L	19,389	144	0.74
M	20,832	191	0.92
N	17,078	149	0.87
O	15,078	165	1.09
**Time of delivery**			
00:00–01:59	23,457	251	1.07
02:00–03:59	24,377	191	0.78
04:00–05:59	24,601	213	0.87
06:00–07:59	23,911	204	0.85
08:00–09:59	22,211	234	1.05
10:00–11:59	23,155	211	0.91
12:00–13:59	22,871	246	1.08
14:00–15:59	22,105	250	1.13
16:00–17:59	21,594	253	1.17
18:00–19:59	21,829	255	1.17
20:00–21:59	21,245	241	1.13
22:00–23:59	22,516	259	1.15

* Pregnancy risk status was included in the multivariable analysis via the inclusion of a number of individual risk factors as model covariates (see ‘Definitions’ section and Table 3). In Table 2, however, it is shown as a single summary variable.

Table 2 describes the characteristics of the group of maternities included in this analysis, and also shows variations in the incidence of PPH according to key characteristics. Chi-squared tests showed that all of these observed variations were highly statistically significant (p < 0.01 for all of the associations shown in Table 2).

The results of the modelling are shown in Table 3 in the form of odds ratios. In the unadjusted data, among those who had low- or medium-risk pregnancies, those who intended a hospital birth were significantly more likely to experience PPH than those who intended a home birth (odds ratio, 2.7, 95% confidence interval (CI) 1.8 to 4.1). After adjustment for the other model covariates, the odds ratio was smaller, but still highly statistically significant at 2.5 (95% CI 1.7 to 3.8). In other words, among those with low- and medium-risk pregnancies, the odds of a woman who had a planned hospital birth experiencing a PPH were 2.5 times the odds of a comparable woman who intended a home birth experiencing a PPH (whether or not she went on to experience a home birth).

**Table 3:** Results of model with postpartum haemorrhage as the outcome

	**Unadjusted odds ratio**	**Adjusted odds ratio**	**95% confidence interval for adjusted odds ratio**	
**Intended place of birth (reference, home)**				
Hospital	***2.7	***2.5	1.7	3.8
**(Suspected) macrosomia? (reference, no)**				
Yes	***4.1	**2.7	1.3	5.6
**Previous baby with birthweight >4500g? (reference, no)**				
Yes	***2.1	*1.6	1.1	2.4
**Mother’s BMI (reference, <30)**				
30-34	***1.4	***1.4	1.3	1.6
**Borderline anaemia? (8.5-10.5g/dl) (reference, no)**				
Yes	***1.4	***1.3	1.2	1.4
**Parity (reference, multipara)**				
Primipara	***1.7	***2.0	1.9	2.2
**Mother’s age at delivery (reference, 30–34)**				
<20	**0.7	***0.6	0.4	0.7
20-24	***0.8	***0.7	0.6	0.8
25-29	**0.9	***0.8	0.8	0.9
35-39	*1.1	*1.2	1.0	1.3
40+	***1.6	***1.6	1.3	2.1
**Mother’s ethnic group (reference, White European)**				
Black African	***1.8	***1.6	1.3	1.9
Black Caribbean	1.2	1.2	0.9	1.5
Mediterranean	0.9	0.8	0.6	1.1
Oriental	***1.7	**1.6	1.2	2.0
South Asian	0.9	1.0	0.9	1.1
Other	*1.3	1.0	0.8	1.2
Missing	0.9	1.0	0.8	1.3
**Current baby’s birthweight (reference, 2500g-3999g)**				
Low (<2500g)	*0.7	**0.6	0.4	0.8
High (4000g+)	***2.4	***2.6	2.4	2.9
				
**Sex of baby (reference, girl)**				
Boy	***0.8	***0.8	0.7	0.8
**Number of ultrasound scans during current pregnancy (reference, 1)**				
0	1.3	1.3	1.0	1.7
2	***1.3	**1.2	1.1	1.3
3	***1.2	*1.1	1.0	1.3
4	***1.7	***1.6	1.4	1.9
>4	***1.7	***1.6	1.2	2.0
Missing	1.0	0.8	0.6	1.2
**Year of delivery (reference, 1988)**				
1989	1.1	1.1	0.9	1.4
1990	**1.4	**1.4	1.1	1.7
1991	1.1	1.1	0.9	1.4
1992	*1.2	*1.3	1.0	1.5
1993	1.2	1.2	0.9	1.4
1994	1.2	1.2	0.9	1.4
1995	***1.5	**1.4	1.2	1.8
1996	**1.4	*1.3	1.1	1.6
1997	***1.6	**1.4	1.1	1.7
1998	***1.6	**1.4	1.1	1.7
1999	***1.7	***1.5	1.2	1.8
2000	***1.7	***1.5	1.2	1.8
**Hospital providing care (anonymised) (reference, H)**				
A	0.9	1.0	0.8	1.3
B	**0.7	**0.7	0.6	0.9
C	0.9	0.9	0.7	1.2
D	***1.6	**1.3	1.1	1.6
E	1.1	*1.4	1.1	1.8
F	1.0	1.0	0.9	1.3
G	**0.8	**0.7	0.6	0.9
I	***1.4	***1.6	1.3	1.9
J	***1.4	**1.3	1.1	1.6
K	*1.2	1.0	0.8	1.2
L	*0.8	0.9	0.7	1.1
M	1.0	1.0	0.8	1.1
N	0.9	0.9	0.7	1.1
O	1.2	1.1	0.9	1.3
**Time of delivery (reference, 10:00–11:59)**				
00:00–01:59	1.2	1.2	1.0	1.4
02:00–03:59	0.9	0.9	0.7	1.1
04:00–05:59	0.9	1.0	0.8	1.2
06:00–07:59	0.9	1.0	0.8	1.2
08:00–09:59	1.2	1.2	1.0	1.4
12:00–13:59	1.2	1.2	1.0	1.4
14:00–15:59	*1.2	*1.2	1.0	1.5
16:00–17:59	**1.3	*1.2	1.0	1.5
18:00–19:59	**1.3	*1.2	1.0	1.5
20:00–21:59	*1.2	*1.2	1.0	1.5
22:00–23:59	*1.3	*1.2	1.0	1.5

*** p < 0.001, ** p < 0.01, * p < 0.05.

As noted in the ‘methods’ section, an argument could be made for including unplanned home births in the analysis, so as a sensitivity analysis the modelling was repeated, including them. The odds ratio and associated confidence interval for intended place of birth were exactly the same as for the model which excluded unplanned home births. Similarly, the analysis was repeated to include unattended births, and again this made virtually no difference to the results (the odds ratio for intended place of birth was 2.5, 95% CI 1.6 to 3.7). We can therefore be confident that the results are not sensitive to the inclusion or exclusion of unplanned home births or unattended births.

Also as noted in the ‘methods’ section, ‘high-risk’ pregnancies were excluded from the main analyses. We did, however, repeat the analysis including ‘high-risk’ pregnancies, and found that the odds ratio for intended place of birth was very similar (OR 2.1, 95% CI 1.4 to 3.4; further details can be provided on application to the authors). Great care was taken to control for pregnancy risk status because the ‘intended a hospital birth’ group contained proportionally more ‘high-risk’ pregnancies than the ‘intended a home birth’ group, and because ‘high-risk’ pregnancies were more likely to have PPHs. Pregnancy risk status was included in the model by treating each high- or medium-risk condition as a separate covariate. Thus, the model controlled for the facts that: (a) the ‘intended a home birth’ group contained a higher proportion of low-risk pregnancies than did the ‘intended a hospital birth’ group, and (b) among those with high-risk pregnancies, those who intended a home birth tended to have different high-risk conditions from those who intended a hospital birth.

## Discussion

PPH is a relatively rare complication; it occurred in just 1% of the deliveries included in this analysis, so even with an odds ratio of 2.5, the absolute risk of an individual woman experiencing this complication is small. Nevertheless, it is a serious complication which is one of the leading causes of maternal death in the UK [26] and worldwide [27], and as such it is important to minimise the risk of its occurrence where possible. This study aimed to compare the risk of PPH between those who intended a home birth at the end of pregnancy (whether or not they went on to experience a home birth) and those who had a planned hospital birth. It found significantly higher odds of PPH among those who had a planned hospital birth than among those who intended a home birth. This raises questions about the safety of hospital birth from the perspective of the mother’s wellbeing.

The incidence of PPH for planned hospital births would be expected to be higher than the incidence for planned home births, because nulliparous women are more likely to experience PPH (see Table 2), and are also more likely to plan a hospital birth [28]. It was therefore not surprising to find that odds ratio was 2.7 before any adjustment was made for confounding. Nevertheless, despite the model controlling for parity and many other known confounders, there remains a greatly elevated risk of PPH for women who have planned hospital births in comparison to those who plan a home birth (odds ratio, 2.5, 95% confidence interval, 1.7 to 3.8).

This result highlights a statistical association between intended place of birth and PPH; it does not prove a causal relationship, nor does it explain *why* the association exists. Previous research has found an association between PPH and procedures including: augmentation of labour, emergency Caesarean section and episiotomy [12,13], all of which were more common among those who intended a hospital birth than among those who intended a home birth in SMMIS. We can therefore speculate that the increased risk of PPH associated with planned hospital birth may be fully or partly explained by the heavier use of these procedures in the hospital setting. In many cases these procedures will be clinically necessary to maximise the safety of mother and/or baby. Further research would be necessary to establish the extent to which they are used when not clinically necessary.

Emergency Caesarean section has become more common in the years since 2000 across Great Britain, and episiotomy rates have remained fairly stable since 2000 [29]. If, therefore, the heightened risk of PPH among those having planned hospital births in 1988–2000 was due to these procedures being more common in hospital than at home, it is unlikely that the situation has changed much since 2000. Given that procedures such as labour augmentation, Caesarean section and episiotomy are associated with an increased risk of the potentially life-threatening PPH, pregnant women and their partners should be advised that, whilst they may be appropriate – and even life-saving - in certain circumstances, such procedures are not risk-free and should not be undertaken without due consideration of the potential risks. Ideally, this information should be provided well in advance of the commencement of labour, to give people time to consider carefully the decisions they might make under different circumstances. These procedures should not be described as ‘safe’ without any caveats; they may be ‘safe’ in terms of obstetric science’s ability to treat any undesirable consequences, but the lay person may understand the term ‘safe’ to mean that there will not *be* any undesirable consequences. Previous research has shown that pregnant women’s definitions of the word ‘safe’ are not always in line with the definitions of maternity care providers [30]. It is important that both understand the word ‘safe’ in the same way, if the rhetoric of informed choice is to become a reality.

The finding that the risk of PPH was lower if a home birth was intended even when ‘high-risk’ births were included in the model raises the question of whether it is necessary for all women with ‘high-risk’ pregnancies to be advised to plan a hospital birth on the grounds of safety. However, given the statistical issues with this model, more research would be needed before drawing any firm conclusions on this point.

### The strengths and limitations of the data

The SMMIS database is extremely useful for the study of pregnancy outcomes by place of birth, because it overcomes many of the problems inherent within other data sources. SMMIS allows the intended place of birth at the end of pregnancy to be derived (see ‘Definitions’ above), rather than relying on the woman’s stated intention in the early stages of pregnancy. Thus, only those who intended a home birth at the *end* of pregnancy are counted as planning a home birth. SMMIS contains over 500,000 observations, so even though fewer than 2% had a planned home birth, the absolute number of planned home births was large enough to give reasonable power to statistical tests. SMMIS allows those who transferred to hospital after an attempt at a home birth to be identified and included in the ‘planned home birth’ group, thus overcoming the bias that would be introduced if the ‘planned home birth’ group contained only those uncomplicated cases which ended in a home birth. SMMIS allows pregnancies to be reasonably objectively classified into different risk categories, thus allowing us to adjust for any bias resulting from planned hospital births containing a disproportionately high number of so-called ‘high-risk’ cases. SMMIS allowed us to control for nearly all of the known risk factors for PPH, many of which were also associated with intended place of birth and would therefore almost certainly have caused problems of confounding had they not been included as model covariates. SMMIS contained information on various socio-demographic characteristics of women giving birth, thus allowing us to control for social and demographic confounders as well as obstetric and medical ones.

Few, if any, existing studies of home birth in the UK can claim to have overcome this many of the problems commonly associated with the study of home birth. Therefore, this study makes a novel and valuable contribution to what was previously known about the safety of home birth.

There were, however, limitations to the SMMIS database. It was collated over the period 1988–2000, the end date occurring more than 10 years ago. However, in the absence of a more recent dataset with the qualities possessed by this one, it represents the most up-to-date, high-quality information available from the UK. The data are also specific to one region of England, so care should be exercised when generalising these results to the UK as a whole. However, the North-West Thames region was large and diverse in terms of geography and demography, so there is no reason to suppose that the results are completely atypical of the rest of the country, with the possible exception of remote rural areas.

It is notoriously difficult to estimate accurately the amount of blood lost during labour and delivery, and the normal method used (visual estimation) has been found to be inaccurate [31]. However, there is no reason to suppose that the estimates in hospital were systematically higher than the estimates at home, so this inherent inaccuracy is unlikely to have biased the relative risk estimates when comparing home and hospital births.

Some potentially useful covariates were not included in the database; most notably whether or not the woman had had a PPH in one or more previous pregnancies. Previous PPH has been found to be a predictor of PPH [12], and it would be reasonable to speculate that women who had had a previous PPH would be more likely to plan a hospital birth than to plan a home birth. If so, the omission of PPH as a covariate will have made the risk associated with planning a hospital birth appear higher. However, because PPH is a rare complication, and because the analysis controlled for predictors of previous PPH (eg previous baby weighing >4500g), it is unlikely that the odds ratio would have been greatly affected had ‘previous PPH’ been included as a covariate.

Because the SMMIS database covered a 13-year period, some women were included in the database more than once, due to having more than one pregnancy during those 13 years. For reasons of confidentiality, these repeated events are not identified on the SMMIS database. There will therefore be clustering effects that were not controlled for in the analysis. This will have affected the study’s conclusions if some women had an underlying propensity towards PPH that was carried through all their pregnancies, and if these women tended to plan for a hospital birth. To assess the extent to which this might be a problem, the model was re-run three times: once based just on women of parity 0 (first-time mothers), once on women of parity 1 and once on women of parity 2. Although the size of the odds ratio varied across these three parity groups, the overall pattern was the same, ie the risk was higher among those who intended a hospital birth. This indicates that the increased risk of PPH among those who intended a hospital birth cannot be explained by uncontrolled clustering effects.

## Conclusions

Pregnant women and their partners who are considering where to give birth should be informed that they may be at higher risk of PPH if they plan a hospital birth than if they plan a home birth. Future research should focus on possible explanations for the significantly higher risk of PPH among those planning a hospital birth, and address the possibility that procedures such as augmentation, emergency Caesarean section and episiotomy are over-used in the hospital setting.

Future research should also attempt to establish whether or not these results also apply to more life-threatening categories of PPH (e.g. >1,500ml of blood lost), and whether the lower incidence of PPH among planned home births translates to fewer cases of PPH-related severe morbidity. Only when these questions are answered will it be possible to make a clear and confident statement about the relative safety of planned home birth in relation to PPH.

## Abbreviations

BMI: Body mass index; CI: Confidence interval; ICD: International classification of diseases; NHS: National health service; NICE: National institute for health and clinical excellence; PPH: Postpartum haemorrhage; RCOG: Royal college of obstetricians and gynaecologists; RHA: Regional health authority; SMMIS: St Mary’s maternity information system database; UK: United Kingdom.

## Competing interests

The authors declare that they have no competing interests.

## Author details

Andrea Nove is a former PhD student from the Division of Social Statistics, University of Southampton, England. Ann Berrington is a Reader in Demography and Social Statistics at the Southampton Statistical Sciences Research Institute and ESRC Centre for Population Change, University of Southampton. Zoë Matthews is Professor of Global Health and Social Statistics at the Centre for Global Health, Population and Poverty, University of Southampton.

## Authors’ contributions

AN was responsible for the study design and statistical analysis, with advice and guidance from AB and ZM. AN wrote the first draft of the paper; AB and ZM provided suggestions and all authors approved the final version.

## Pre-publication history

The pre-publication history for this paper can be accessed here:

http://www.biomedcentral.com/1471-2393/12/130/prepub
